# Impact of fall armyworm (*Spodoptera frugiperda*) (J.E. Smith) on small-scale maize farmers and its control strategies in the Limpopo province, South Africa

**DOI:** 10.4102/jamba.v13i1.1016

**Published:** 2021-10-27

**Authors:** Mankwana C. Makgoba, Phumudzo P. Tshikhudo, Livhuwani R. Nnzeru, Rudzani A. Makhado

**Affiliations:** 1 Centre for Sustainable Agriculture, Rural Development, and Extension, Faculty of Natural and Agricultural Sciences, University of the Free State, Bloemfontein, South Africa; 2 Department of Agriculture, Land Reform and Rural Development, Directorate Plant Health, Division Pest Risk Analysis, Arcadia, Pretoria, South Africa; 3 Department of Forestry, Fisheries and the Environment, Directorate Biosecurity, Cape Town, South Africa; 4 Department of Biodiversity, University of Limpopo, Sovenga, South Africa

**Keywords:** Quarantine pest, *Spodoptera frugiperda*, fall armyworm, maize, small-scale farmers

## Abstract

South Africa experienced major outbreaks of fall armyworm (*Spodoptera frugiperda*) (J.E. Smith) (Lepidoptera: Noctuidae), causing direct damage by feeding on both vegetative and reproductive parts of host plant. The study was conducted to determine the level of impact of fall armyworm on small-scale maize famers after the outbreak of fall armyworm and their control strategies at Ga-Mashashane and Mankweng villages in the Limpopo province. Semi-structured questionnaire was designed to gather information on the damage caused by fall armyworm, economic impact on the local market and control measures on fall armyworm. Using a snowball sampling procedure, 63 small-scale maize farmers from the two villages of the Limpopo province, South Africa, were randomly selected for this study. The results showed that all participants could correctly identify the fall armyworm and reported it as the most important maize pest during 2016–2017 cropping season. The maize yield loss experienced by affected farmers in the 2016–2017 cropping season was slightly lower as compared with the 2015–2016 harvest. These farmers used pesticides as a control measure for fall armyworm. Fall armyworm has become a major pest in South Africa and the tackling of fall armyworm by small-scale farmers and averting yield losses is thus critical. This study contributes to the knowledge on fall armyworm management by small-scale farmers, which is essential to enhancing food security.

## Introduction

Maize (*Zea mays* L.) is one of the most significant staple foods to many African communities, including South Africa. Over 200 million people in Africa are dependent on maize for food security (Abrahams et al. 2017a). A study of this nature would be futile without a proper review of understanding of the global impact caused by fall armyworm on host plants. The African continent experienced major outbreaks of fall armyworm (*Spodoptera frugiperda*) (J.E. Smith) (Lepidoptera: Noctuidae) attacking maize crop, which is difficult to control. *Spodoptera frugiperda* is widely distributed in America, causing the most serious damage to about 80 different commercial crops, including maize, rice, sorghum, sugarcane, cabbage, beet, groundnut, soybean, alfalfa, onion, pasture grasses, millet, tomato, potato and cotton (Day et al. 2017). Sweet corn is the vegetable crop that is most vulnerable amongst others. In Uganda, this pest attacked crops such as cotton, sugar cane, banana and vegetables (Tajuba 2017). The insect pest also prefers grasses, in which the field crops that are often damaged include barley, bermudagrass, buckwheat, clover, oats, peanuts, ryegrass, sugarbeets, sudangrass, soybeans, sugarcane, timothy, tobacco and wheat. Fall armyworm can cause damage to host plant species such as *Sorghum halepense*, bentgrass, crabgrass, johnsongrass, nutsedge, pigweed, sandspur and *Cenchrus tribuloides* (Barlow 2009).

*Spodoptera frugiperda* primarily causes damage by feeding on both vegetative and reproductive parts of host plant. Fall armyworm larvae damage crops through defoliation. The insect pest can cause a serious damage to the fruit, resulting in premature drop and fruit rot on tomato and pepper (Abrahams et al. 2017a). The first instar larvae start feeding nearby to the ground. The larvae eat the leaf tissue from one side by leaving the opposite epidermal layer intact. At the second or third instar stage, larvae start to cause holes in leaves and consume from the edge of leaves on the inward. Holes on the maize crop are formed because of the feeding of folded leaves. Rows of three to four small to large holes are observed across the leaf at the time when leaves grow out (Abrahams et al. 2017b). The densities of larval always reduce to 1–2 per crop because of the behaviour of cannibalistic. The densities of 0.2–0.8 larvae per crop occurring at the late whorl stage can decrease yield by 5% – 20% (Capinera 2017). The older larvae can cause defoliation by leaving the crop with a ragged and torn up appearance. These larvae can also cause damage by burrowing into maize tassels and ears (Abrahams et al. 2017b). Usually, the young larvae hide in the funnel of maize during the day and at night it emerges to consume the leaves (Day 2017).

In Zambia, a loss of 40% was reported as the latest outbreaks with an estimation of 124,000 ha of maize being attacked (Kansiime et al. 2019). In 2017, fall armyworm was reported to attack about 9000 ha of maize in Malawi (Wilson 2017). Fall armyworm can breed throughout the year in Brazil and about US$600 million a year is required for controlling this pest (Wilson 2017). Based on the study conducted by CABI (2017), fall armyworm damages resulted in losses of maize in Ghana and Zambia at 45% and 40%, respectively, in July 2017. Cruz (1999) reported a yield loss of approximately 34% under maize production. In most cases, yield losses are because of larval defoliation. Yield reduction in rice was correlated with larval density within the maize fields (Pantoja et al. 1986).

In a laboratory research conducted by Sparks (1979), the first three instars can cause about 2% of damage, whilst the fourth instars causes about 75% of damage to the total yield. In field trials, fall armyworm caused a significant loss in yield (17%), when 20% of maize plants were infested with an egg mass (Cruz & Turpin 1983). In the United States of America, major outbreaks of fall armyworm occur in warmer seasons as results of adults’ dispersal from the northern part of America (Cruz & Turpin 1983). Fall armyworm was reported to cause the annual average yield loss of $60 m between 1975 and 1983 in the United States of America (Ellis 2005), whereas in Brazil the loss was more than US$400 million damage annually (IITA 2016). It was estimated that Brazil spends about US$600 mn annually controlling the fall armyworm outbreaks (Wild 2017). Fall armyworm impacts international trade because of its rapid rate of introduction, spread and establishment (Maynard et al. 2004; FAO 2004; IPPC 2004). Thus, countries in Europe, North Africa and Asia manage the pest through development of strict phytosanitary import requirements. For example, the first consignment of roses exported from Africa contaminated with fall armyworm was intercepted in Europe during 2017 (Day 2017).

Fall armyworm caused more damage to maize plant than other species falling within the same genus present in Africa (Du Plessis et al. 2018). It is difficult to control fall armyworm once the population is high. Fall armyworm is a cosmopolitan of the maize plant. It consumes all stages of maize development, mostly the whorl of young plants until 45 days old (Cruz 1999).

The first detection of fall armyworm in the African continent was recorded in Nigeria at the beginning of 2016, where it later spread to several western and central parts of African continent by April 2016 (Cock et al. 2017; Goergen et al. 2016). In South Africa, the Department of Agriculture, Land Reform and Rural Development (DALRRD) confirmed the presence of *S. frugiperda* in February 2017 by positive identification of caterpillars and adult moth, where it was also published on the International Plant Protection Convention (IPPC)’s portal in terms of South African international pest reporting obligations (DALRRD 2017).

The presence of fall armyworm in South Africa was confirmed in provinces such as the Limpopo, Gauteng, North West, Mpumalanga, KwaZulu-Natal, Free State and Eastern Cape (DALRRD 2017). Fall armyworm is still new to African countries and as a result, natural enemies are still rare for classical biological control (FAO 2017). This pest is difficult to control as the maize plant mature: the caterpillars feed inside the leaf whorl outside the reach of chemicals (pesticides). It poses a serious economic risk to maize farmers in South Africa by causing direct damage to maize crop resulting in major yield losses. In South Africa, the production of maize contributes the biggest component within grains. About 43% of maize production is for white maize and 57% is for yellow maize. Free State and North West provinces are the main producers of maize, contributing 60% to both white and yellow maize (DALRRD 2016).

Fall armyworm occurs in most of the production areas of maize within the Republic of South Africa. However, the infestation of this insect pest does not occur in a larger population in some parts of the provinces, such as Gauteng, North West, Free State and Eastern Cape. The high infestation occurs in the Limpopo province and the Umkhanyakude District Municipality in the Kwazulu–Natal province. The infestation rate seems to be increasing in the Mpumalanga province. In Northern Cape and Western Cape provinces there were no reported outbreaks (DALRRD 2018). During 2016, after its invasion to African countries, fall armyworm has been perceived as a threat to the production of grain on the Southern African Development Community (SADC) region.

This study, therefore, was initiated to (1) determine the level of impact of fall armyworm outbreak on small-scale maize famers including maize yield, (2) determine the knowledge of farmers about fall armyworm by farmers and (3) control strategies practised in the Limpopo province, South Africa. In subsistence maize production, small-scale farming is primarily grown for consumption by the farmers and their family. However, if there is a surplus of food it might be sold, but that is not common in villages of the Limpopo province of South Africa.

## Methods

### Study area

**FIGURE 1:**
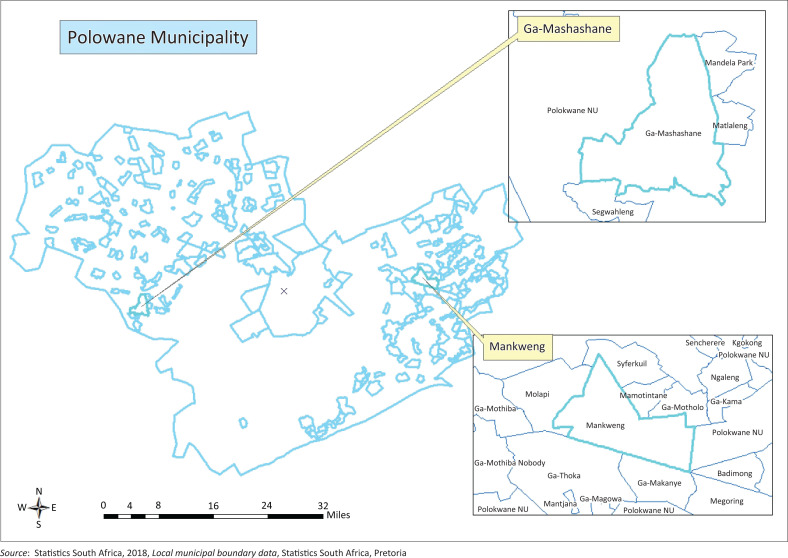
Geographical locations of Ga-Mashashane and Mankweng at Capricorn District, Limpopo province, South Africa.

The study was conducted in 2016 at the villages of Mankweng and Ga-Mashashane in the Polokwane Manicipality of the Limpopo province, South Africa (Figure 1). Maize is a crop grown throughout the rural areas of Capricorn District as a staple food. The climate of these areas are characterised by summer rainfall. The climate of the Capricorn district is predominately subtropical. The highest temperature occurs during December and January, with daily average maximum temperature ranging from 28 °C to 36 °C. The rainfall normally occurs between November and March. The average annual rainfall ranges from 454 mm to 478 mm. These areas are characterised by level to undulating plans and rich fertile soils (Ditau Geo-Informatics Solutions 2016).

### Data collection and analysis

In this study, field surveys were conducted at the villages of Mankweng and Ga-Mamabolo to gather information on the impact of fall armyworm to small-scale maize farmers and its control strategies. A semi-structured questionnaire was used to gather information from 63 small-scale farmers sampled in the selected villages of the Limpopo province. The questionnaire was used to interview the respondents. Small-scale farmers were sampled using a snowball sampling method. The snowball is a non-probability sampling method and was applied in this study to allow sampled farmers to recruit other farmers in the village to participate in the study.

A questionnaire was used to capture primary information on interviewees gender, age, home language, educational level; farmers experience in maize production; status of fall armyworm; damage caused by fall armyworm; economic impact of fall armyworm to the local market industry; and control measures of fall armyworm by farmers. Camera images were also captured to provide evidence of the existence of fall armyworm and its control measures. Completed questionnaires from the respondents were gathered and analysed.

Collected data were captured in Ms Excel 365 and analysed using Statistical Package for the Social Sciences (SPSS). The SPSS was used because it is good for analysing social survey data. Data were analysed quantitatively using descriptive statistics and presented using frequency tables, graphs and charts.

## Results and discussion

The results on the demography of farmers, status of fall armyworm, damage caused by fall armyworm, economic impact of fall armyworm on the local market industry and control measures were presented and discussed as follows:

### Respondents’ characteristics

Maize farming plays an important role in supplying food to rural people and creation of seasonal employment opportunities. The prevalence of female headed households in most rural areas in the Limpopo province of rural South Africa has an impact on household and community livelihood strategies (Baloyi 2011). The study found that females were the most dominating farmers (62%) compared with males (38%) with regard to maize farming. This could be because of the fact that most of households in rural areas are headed by women. They fully contribute to family farming with their labour and knowledge of crop production. Our findings agree with De Groote et al. (2020), who found that women are more involved in farming than men. The poorer women in most rural villages practice farming more out of desperation (Hirschmann & Vaughan 1983). This suggests that women at the villages rely on maize farming as a source of food and income. This could also play a big part in contributing to the rural economy (Damisa & Yohanna 2007, Butt et al. 2010, Singh et al. 2020). In terms of age, the study indicates that old-age people have passion for agriculture when compared with younger age group, which implies that the youth lack interest in farming as 92.06% of farmers were above 35 years of age. Youth perceived that farming is not profitable and thus choose to venture into other businesses than smallholder farming. Increases in both the percentage and absolute numbers of elders, originally observed in industrialised countries are now a concern for a growing number of developing countries (Woodsong 1994). There is concern that the rural concentration of elders may have negative consequences for agricultural production in terms of understanding and adopting new agricultural policies (Balezentis et al. 2020).

In terms of home language and the education, the majority of the respondents (94%) speaks Northern Sotho. Other languages spoken by few include XiTsonga, IsiZulu, IsiNdebele at 3%, 2% and 2%, respectively. The majority (92%) of the maize farmers received high school education. The remaining 5% had university education whilst 3% had Further Education and Training (FET) offered in technical, community and private colleges. Farming requires technical knowledge through the experience of involvement in farming (Ozowa 1995), but the theoretical knowledge of farming is necessary, that is why the Limpopo Provincial Department of Agriculture extension officers are engaged with farmers from time to time to provide them with theoretical knowledge regarding quarantine pest such as fall armyworm. Education plays a positive role on agricultural productivity in the presence of rapid technical change because it assists farmers to adjust more readily to the new opportunities provided by technological innovations (Reimers & Klasen 2013). On the one hand, education is important to the improvement of agricultural productivity because it improves the farmer’s knowledge. On the other hand, non-formal education affords the farmer hands-on training and better methods of farming, whilst informal education keeps the farmer abreast of changing innovations and ideas and allows farmer to share their experience (Oduro-Ofori et al. 2014).

### Farmers experience in maize production

About 10% of maize farmers were farming with maize for 11–15 years, 43% were farming with maize for 16–20 years, 38% were farming with maize for 21–25 years, 3% were farming with maize for 26–30 years, 5% were farming for 1–5 years and 2% were farming for 31–35 years (Table 1). This implies that majority of the farmers have 11–25 years of experience in terms of planting maize. Farming experience assist in pest identification, which thus becomes easier to come-up with control measures for that particular identified pest. Farmers’ knowledge has an important role to play in bringing about sustainable innovations in agriculture (Rajendran et al. 2016; Stuiver, Leeuwis & Van der Ploeg 2004).

**TABLE 1:** Years of farming with maize around the area.

Number of years in maize farming	Frequency	Frequency, %
1–5 years	3	4.76
11–15 years	6	9.52
16–20 years	27	42.85
21–25 years	24	38.09
26–30 years	2	3.17
31–35 years	1	1.58

### Status of fall armyworm

**FIGURE 2:**
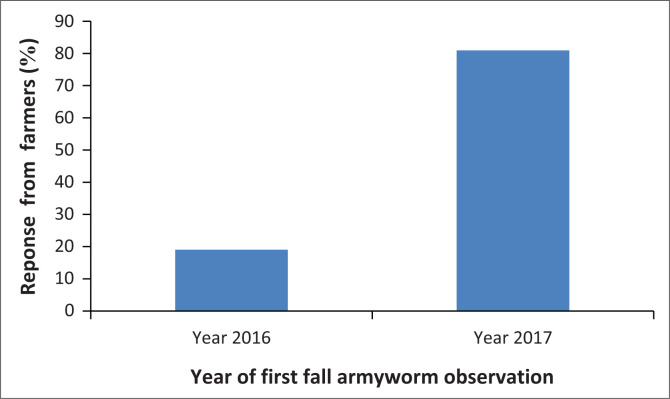
Farmers response on the years for first fall armyworm observation.

The results of the study revealed that fall armyworm is present in all the farmers maize fields. Considering that maize is a staple food in the area, the occurrence of fall armyworm threatens maize production and food security in the study area. The study found that 19% of small-scale maize farmers started to observe the fall armyworm at their maize fields during 2016 planting season. However, the majority of the farmers (81%) became aware of fall armyworm damage during the 2017 planting season (Figure 2).

The study found that all the 63 small-scale maize farmers who participated in this study were able to identify fall armyworm on the maize plant. All maize farmers were able to detect and provide with the correct description of fall armyworm. The farmers were able to distinguish the worm from other insect pest occurring at their maize fields. As also found in Ethiopia and Kenya by Kumela et al. (2018), small-scale maize farmers in the study have the ability and capability to identify insect pest in all the life stages including eggs on the infested maize crop.

The results of the study further revealed that fall armyworm is present in all the farmers maize fields. The respondents further indicated that the extension official from the Limpopo Provincial Department of Agriculture and Rural Development assisted with the accurate identification of the worm at the maize fields. Specimens of pests were sent to the National DALRRD for further inspection and identification at the diagnostic laboratories for official confirmation. The occurrence of fall armyworm threatened maize production and food security in the sampled villages.

### Damage caused by fall armyworm and rate of spread

**FIGURE 3:**
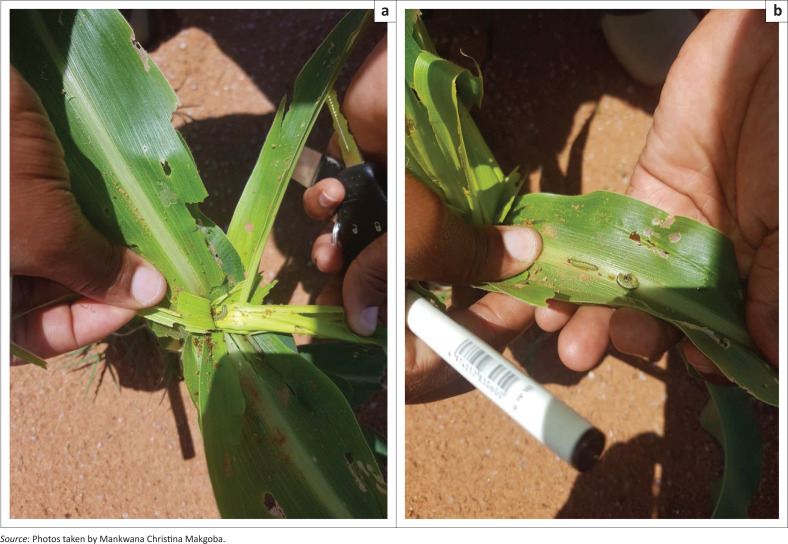
Fall armyworm causing damage on maize plant at Maltapa Dryland Farm.

Regarding the damage of fall armyworm on maize plant, 57% of the farmers observed that the worm attacked and damaged the leaves and stems of maize plant and 43% observed that the worm eats the maize plant from inside (see Figure 3). This study concurs with other studies, which shows that the larvae cause damage at all vegetative stages of maize plant (e.g. De Groode et al. 2020; Kumela et al. 2018). The female fall armyworm produce 900–1000 eggs in 30 days, making it a successful colonizing species (Johnson, 1987). The larvae defoliate the whole maize plant if they are abundant (Barlow 2009). They consume the leaf tissue from side by causing a layer intact in the epidermal (Abrahams et al. 2017a).

There are different ways in which fall armyworm attacks the maize plant. Most of the farmers (57%) observed holes on the maize leaves made by the worm, whilst 21% observed that the worm damages the corn ears, and 22% observed damage on maize heads (Table 2). According to Abrahams et al. (2017a), fall armyworm larvae caused damage by burrowing into maize ears. The larvae also cause holes in the leaves by consuming from the edge of leaves on the inward. During the night, the larvae emerge to consume the leaves (Day 2017).

**TABLE 2:** Fall armyworm damage on maize plant.

Damaged parts	Frequency, %
Leaves	57
Ears	21
Head	22

About 92% of the farmers observed that the rate of spread and reproduction of fall armyworm was high, whilst 8% observed that there was new reproduction every 3 days. According to the study conducted by Capinera (2017), the densities of larvae reduce to 1–2 per crop because of the characteristics of cannibalistic. Normally the densities of 0.2–0.8 larvae per crop occurring at the late whorl stage can cause a reduction on yield by 5% – 20% (Kumela et al. 2018). In addition, maize farmers in Ethiopia experienced that infestation of maize by the insect pest was on a range of about 24% – 39%. In other countries such as Kenya, the infestation was about 38% – 54% (Kumela et al. 2018).

**FIGURE 4:**
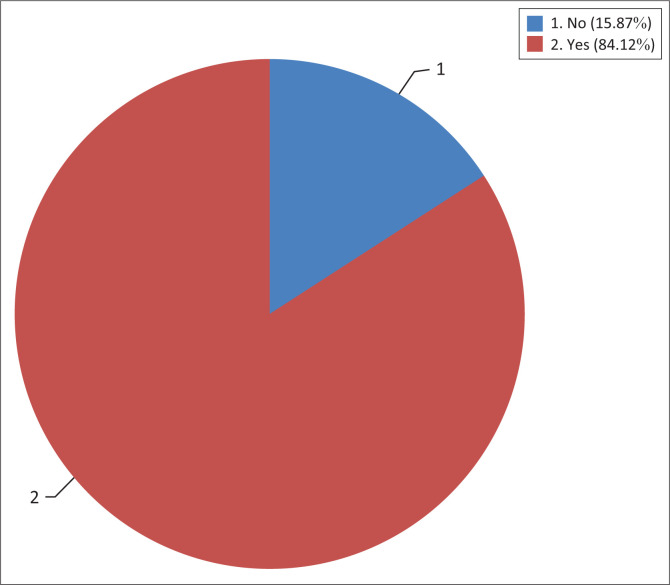
Farmers’ perception (%) on the impact of fall armyworm on maize varieties planted.

About 15.87% of the maize farmers have a perception that fall armyworm did not affect all the varieties of maize. About 16% of those who perceive fall armyworm not to affect all varieties indicated that popcorn varieties (H2072) are resistant to fall armyworm, 2% perceived that organic hybrids are resistant as well and the other 2% also perceived that Bt maize are resistant cultivars. The majority (84%) have a perception that the worm affects all varieties of maize (Figure 4). This might be because a majority of farmers (71%) in the study area planted Pannar 667 and fall armyworm attacked the maize variety, whilst 10% planted border king white maize, which was also attacked by the worm. Previous studies reported that yield loss of modern varieties because of fall armyworm was not significant as they received adequate fertilizers or planted on rich soils (Baudron et al. 2019; Houngbo et al. 2020).

This reveals that farmers might be farming with the same product but the skill, experience and problems each farmer encounters at their daily lives may be different. According to Day et al. (2017) in the United States of America, fall armyworm caused damage to about 80 different commercial crops, including maize, rice, sorghum, sugarcane, cabbage, beet, groundnut, soybean, alfalfa, onion, pasture grasses, millet, tomato, potato and cotton. In accordance with the study of Luis and Robert (1998), the field maize of corn, which matures at a later stage attracts the moth for laying its eggs.

### Economic impact of fall armyworm

**FIGURE 5:**
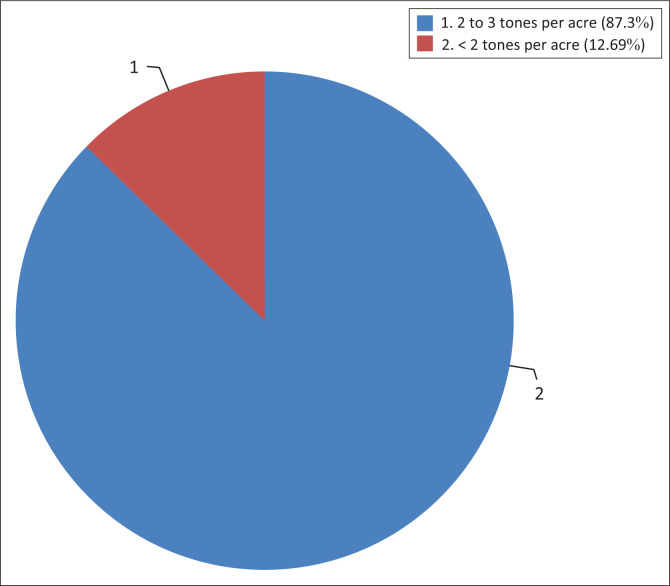
Farmers perception (%) on the amount of maize (ton/ha) produced before and after fall armyworm infestation.

Fall armyworm cause direct damage to maize production, which also affect food production and economic returns to farmers. The maize harvest decreases because of physical damage by the fall armyworm. With regard to replanting of the maize, all the farmers never replanted the maize during the growing season. The majority of the farmers with an average of 87% used to produce 2–3 tons per acre before the occurrence of fall armyworm, whilst the average of 13% produced less than 2 tons per acre (Figure 5). Before the outbreak of the fall armyworms, the production was estimated to be at 3–4 tons per acre. It was further found that small-scale farmers normally practice their farming on a small land without even using advanced technologies, which could be the reason they do not produce many tons.

Based on the study conducted by Day et al. (2017), fall armyworm damages resulted in losses of maize in Ghana and Zambia accounted for 45% and 40%, respectively, in July 2017. Cruz (1999) reported a yield loss of approximately 34% under maize production. In most cases, yield losses are because of larval defoliation. For instance, yield reduction in rice was correlated with larval density within the maize fields (Pantoja et al. 1986).

The respondents further indicated that the outbreak of fall armyworm caused the impact on local market, as it leads to increased price on the purchase of maize seeds and maize meal. Fall armyworm affected their livelihoods as it reduced their yields and increased maize production costs. In Nicaragua, Hruska and Gould (1997) demonstrated a positive relationship between yield losses and levels of fall armyworm infestation. As also indicated by Day et al. (2017), fall armyworm impact the international trade, as trade between countries carries the risk of introduction of pests within countries where the pest are not occurring (the risk is on agricultural plant and plant products). This risk is managed by Europe, Asia and North Africa countries through handling requirements and conditions on exports from fall armyworm affected countries, through the implications cost for the exporters (Day et al. 2017).

### Fall armyworm control measures

**FIGURE 6:**
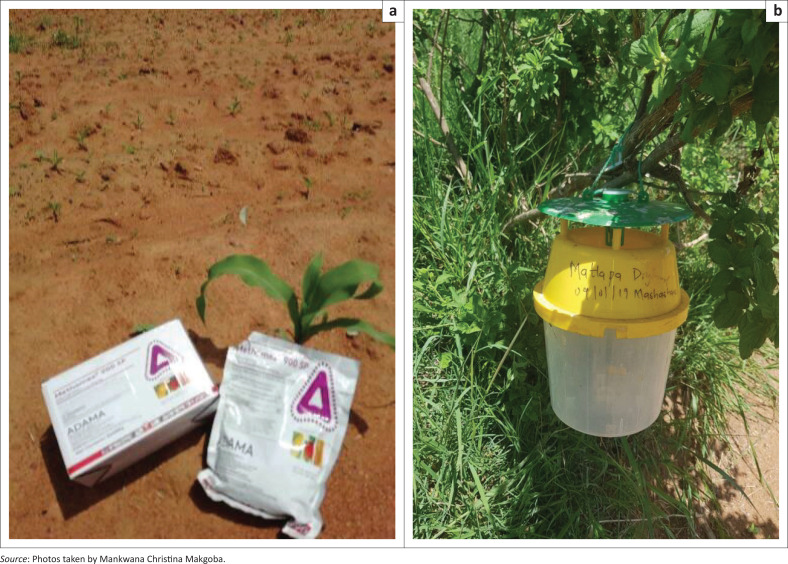
(a) Methomex 900 SP chemical used to control fall armyworm (2 X 500 g); (b) Fall armyworm trap at Matlapa Dryland maize field farmers.

The results of the study revealed that farmers apply chemicals to control the worm at their maize fields. The farmers applied Methomex 900 SP and net contents: 2 X 500g (Figure 6). The chemicals were supplied by the provincial Department of Agriculture and Rural Development for free of charge to farmers. These chemicals seem to be less effective in controlling matured fall armyworm, especially when repeatedly applied. As indicated by Geoergen (2016), pesticides are not effective for older larvae. In Latin America registered pesticides are also recommended for control measures (Day 2017). In Brazil, farmers also use repeated applications of insecticides, on average five sprays were required for the control of fall armyworm in maize (Kumela et al. 2018). Massive use of pesticides on maize crops was applied by farmers in Kenya and Ethiopia to control the worm. Cultural methods such as maize intercropping with common beans, handpicking and killing of caterpillars, application of wood ashes are applied in Africa to control fall armyworm (Kumela et al. 2018).

With regard to the application of control measures for fall armyworm, 29% of the farmers apply the chemical after planting, 33% apply the chemical immediately they observe that the worm is present at their maize fields, whilst 38% apply the chemical before planting (Table 3). The same was observed in other African countries such as Benin, Ghana, Zambia, Kenya and Ethiopia (Baudron et al. 2019; Houngbo et al. 2020; Kumela et al. 2018). However, the most effective time of application was after planting. Farmers also use traps to monitor the fall armyworm level of infestation in the area. With reference to whether the application of control measures were effective or not, all the farmers confirmed that the chemical was effective in terms of controlling the worm. Agricultural extension officers were key sources of information for farmers; hence, it is not surprising that access to extension information on fall armyworm was positively and significantly associated with the adoption of pesticide. All the farmers experienced that once the fall armyworm occurs at the maize field, it is a disaster because it becomes difficult to control.

**TABLE 3:** Period of pesticide application.

Period of pesticide application	Frequency	Frequency, %
After planting	10	28.57
Immediately after detection	45	33.33
Before planting	6	38.09

About 90% of the respondents prevent the worm by applying chemical control during planting, 6% conduct scouting at the maize field with the aim of identifying if the worm is present and to estimate the density of the worm population if it is occurring. Monitoring, surveillance and scouting are critical activities necessary for successful implementation of an effective control programme and the study confirmed that smallholder farmers are aware of IPM applicable to the fall armyworm. Spraying should be applied when the caterpillars are smaller than 1 cm long, bigger caterpillars crawl deep into the leaf whorls of maize plants and it becomes difficult to reach them with agrochemical sprays (DALRRD 2018). According to Barlow (2009) sweetcorn requires four applications per week during the silking and ear development stages. If the level of damage reaches economic threshold control should be carried out by applying insecticides. The study conducted by Day (2017) recommended that spraying should be performed during dawn as it is more effective that time.

## Conclusion

This study concluded that fall armyworm is a serious threat to maize production, which rural people depends on as a staple diet. High outbreak of the worms affects maize production, which could negatively affect the local economy. It could further threaten food security. Although small-scale farmers are aware of fall armyworm morphology and damage, its control measures varies based on support provided and individual farmer capacity to control the worm. It is further concluded that the Limpopo Department of Agriculture and Rural Development should continue to support small-scale farmers to prevent and control the outbreak of fall armyworm.

It is recommended that the pesticides provided to small-scale farmers should be registered by the DALRRD. Awareness and promotions on potential quarantine pests should be enhanced to small-scale farmers. Training and awareness regarding application and knowledge of chemical control products of quarantine pest should be provided to small-scale farmers. It is also important to note that over-utilisation of similar active ingredients may lead to resistance by the target pest. Future studies should focus on maize pests risk analysis and control measures as a mechanism to enhance food security and rival of local economy.
